# Symptoms and health-related quality of life in Japanese patients with primary biliary cholangitis

**DOI:** 10.1038/s41598-018-31063-8

**Published:** 2018-08-22

**Authors:** Minami Yagi, Atsushi Tanaka, Masanori Abe, Tadashi Namisaki, Hitoshi Yoshiji, Atsushi Takahashi, Hiromasa Ohira, Atsumasa Komori, Satoshi Yamagiwa, Kentaro Kikuchi, Tetsuya Yasunaka, Akinobu Takaki, Yoshiyuki Ueno, Akira Honda, Yasushi Matsuzaki, Hajime Takikawa

**Affiliations:** 1 Department of Medicine, Teikyo University School of Medicine, Tokyo, Japan; 2 Department of Gastroenterology and Metabology, Ehime University Graduate School of Medicine, Ehime, Japan; 3 Third Department of Internal Medicine, Nara Medical University, Nara, Japan; 4 Department of Gastroenterology, Fukushima Medical University School of Medicine, Fukushima, Japan; 5 Clinical Research Center, National Hospital Organization (NHO) Nagasaki Medical Center, Nagasaki, Japan; 6 Department of Gastroenterology and Hepatology, Niigata University Graduate School of Medical and Dental Sciences, Niigata, Japan; 7 The Fourth Department of Internal Medicine, Teikyo University Mizonokuchi Hospital, Kanagawa, Japan; 8 Department of Gastroenterology and Hepatology, Okayama University Graduate School of Medicine, Dentistry, and Pharmaceutical Sciences, Okayama, Japan; 9 Department of Gastroenterology, Faculty of Medicine, Yamagata University, Yamagata, Japan; 10 Division of Gastroenterology and Hepatology, Department of Internal Medicine, Tokyo Medical University Ibaraki Medical Center, Ibaraki, Japan

## Abstract

Although patients with primary biliary cholangitis (PBC) experience a variety of symptoms that could impair health-related quality of life (HRQOL), no studies regarding symptoms and impact of PBC on HRQOL have been performed in Asian countries. Herein, we aimed to evaluate symptoms and HRQOL in Japanese PBC patients. We performed a multicenter, observational, cross-sectional study. The PBC-40 and the short form (SF)-36 were used as measures of symptoms and HRQOL. Four-hundred-ninety-six patients with PBC were enrolled. In the PBC-40, the average score was highest in the emotional domain, followed by the fatigue domain. The HRQOL measured using SF-36 was also impaired, especially in the physical and role-social components. After adjustments of variables, female sex, younger age at diagnosis, and lower serum albumin level were independently associated with fatigue scores, while a longer follow-up period and lower serum albumin levels were associated with itch scores.

## Introduction

Primary biliary cholangitis (PBC) is a chronic cholestatic liver disease characterized by immune-mediated destruction of small and medium-sized intrahepatic bile ducts^1,2^. PBC affects predominantly women in their fifth and sixth decades of life, with a male to female ratio of 10:1. Originally, PBC was an insidiously progressive disease, with a high possibility of progression to liver failure and mortality. In current daily practice, however, the diagnosis of PBC can be determined at very early stage with the elevation of cholestatic enzymes and detectable anti-mitochondrial antibodies (AMA). Progression to cirrhosis can be avoided in most cases with treatment intervention with ursodeoxycholic acid (UDCA)^3^. As a result, a substantial proportion of patients with PBC lack evident symptoms such as jaundice and physicians tend to regard their patients as asymptomatic^4,5^.

Nevertheless, patients with PBC experience a variety of subjective symptoms, such as fatigue and pruritus^6–8^, which could severely impair health-related quality of life (HRQOL). Therefore, an improvement of HRQOL should be defined as another important clinical outcome. To measure the HRQOL of patients with PBC in an objective and reproducible manner, the PBC-40, a disease-specific measure of symptoms and HRQOL was developed^9^ and are widely used in clinical studies^10–19^ and clinical trials^20,21^ in Europe and the United States, and especially in the United Kingdom (UK). In addition, the PBC-40, originally developed in English, has been translated into other languages and validated in other European countries^22–24^. However, to date, the measure and characterization of HRQOL in patients with PBC have not been reported in Asian countries. Since differences in ethnic, geographical, cultural, and social background may critically influence symptoms and HRQOL, experience in Europe cannot be extrapolated to PBC patients in other regions of the world.

We have previously developed the Japanese version of the PBC-40 and measured the HRQOL of a small number of Japanese PBC patients^25^. In the current study, we employed a larger cohort form a multicenter collaboration in Japan, and measured and characterized HRQOL of Japanese PBC patients.

## Results

### Study participants

A total of 496 patients with PBC were enrolled, and clinical characteristics at enrolment were shown in Table 1. Males were 10.7% and mean age was 66.0 years old at time of participation. The median of follow-up period after diagnosis was 6.2 years. Although all these patients were currently at the outpatient clinic, one or more clinical events (jaundice, ascites, oedema, hepatic encephalopathy, oesophageal or gastric varices, and hepatocellular carcinoma) were present in 49 (11.8%) of the patients. Treatment with UDCA and bezafibrate was reported in 384 (88.3%) and 100 (23.0%) of patients, respectively. Histological findings at diagnosis were obtained from 285 patients (57.4%) and Scheuer’s staging (I/II/III/VI) was 174/78/27/6, respectively. As comorbidities, Sjögren’s syndrome, chronic thyroiditis, and rheumatoid arthritis exist in 84 (16%), 65 (13%), and 35 (7%) patients, respectively. Medications for pruritus were administered in 24 (5%) patients, including anti-allergic drugs in 20 and nalfurafine hydrochloride in 7 (both in 3).

**Table 1:** Clinical characteristics of study participants.

Variables	
Sex (male, %)	53 [10.7]
Age at diagnosis (years old)	58.3 [11.2]
Age at participation (years old)	66.0 [9.9]
Follow-up period (years)	6.2 [1.9–12.4]
AST (U/L)	27 [22–34]
ALT (U/L)	19 [14–26]
Alkaline phosphatase (xULN ^*^)	0.83 [0.65–1.06]
≥1.50 (n, %)	37 [8.1]
Bilirubin (mg/dL)	0.62 [0.5–0.8]
≥2.0 mg/dL (n, %)	13 [2.8]
Albumin (g/dL)	4.1 [3.9–4.3]
≤3.5 g/dL (n, %)	33 [7.6]
Clinical events (present, %)	49 [11.8]
Use of UDCA (yes, %)	384 [88.3]
Use of bezafibrate (yes, %)	100 [23.0]

Continuous variables are shown as mean [standard deviation] if they are normally distributed and as median [interquartile range] if not.

^*2^ULN, upper limit of normal.

### PBC-40 scores

**Figure 1:**
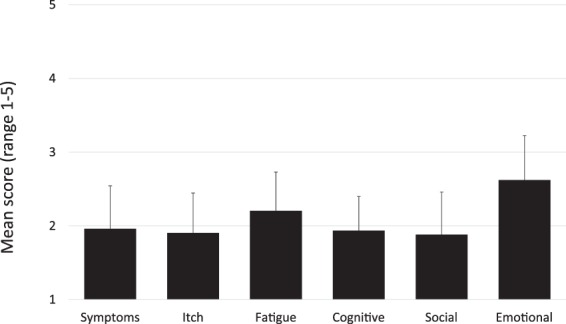
The average score of each PBC-40 domain in Japanese PBC patients (n = 496) is shown. The score was ranged from 1 to 5. The mean score was 2.0 in symptoms, 1.9 in itch, 2.2 in fatigue, 1.9 in cognitive, 1.9 in social, and 2.6 in emotional.

**Figure 2:**
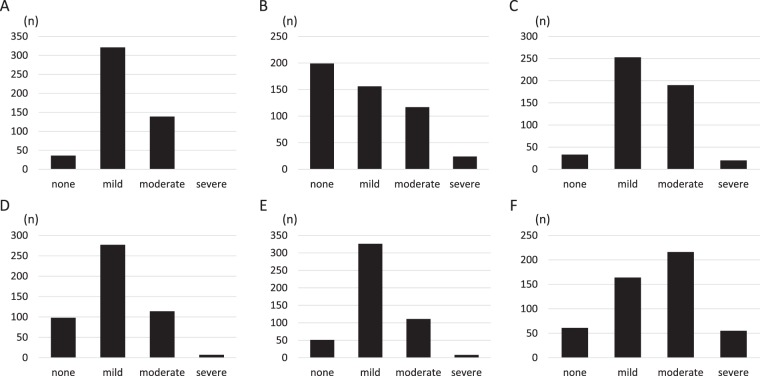
The number of patients in the four categories of severity, “none”, “mild”, “moderate” and “severe”, of each domain is shown. Four categories were defined as follows; “none” if the score was 0, “mild” if the score was between 0 and the one-third of the full score, “moderate” for scores between one-third and the two-thirds of the full score, and “severe” for scores between the two-thirds of the full score and the full score. (**A**) symptom. (**B**) itch. (**C**) fatigue. (**D**) cognitive. (**E**) social. (**F**) emotional.

PBC-40 scores were obtained from all 496 participants. The average score of each domain was 2.0 in symptoms, 1.9 in itch, 2.2 in fatigue, 1.9 in cognitive, 1.9 in social, and 2.6 in emotional, being highest in the emotional domain followed by the fatigue domain (Fig. 1). The distribution of severity for each domain is shown in Fig. 2A–F. The number (proportion) of “moderate” and “severe” in each domain was 139 (28.0%) in symptoms, 141 (28.4%) in itch, 210 (42.3%) in fatigue, 121 (24.3%) in cognitive, 119 (24.0%) in social, and 271 (54.6%) in emotional.

### SF-36 scores and association of PBC-40 with SF-36

**Figure 3:**
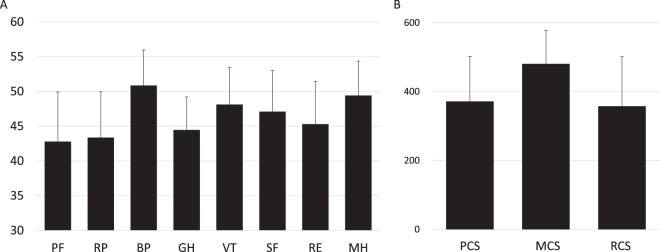
(**A**) The average score of each SF-36 domain in Japanese PBC patients (n = 496) is shown. The score in each domain was 42.8 (PF), 43.4 (RP), 50.9 (BP), 44.5 (GH), 48.1 (VT), 47.1 (SF), 45.3 (RE), and 49.4 (MH). In SF-36, the score 50 was defined as the average of healthy Japanese individuals. PF: physical functioning, RP: role-physical, BP: bodily pain, GH: general health perception, VT: vitality, SF social functioning, RE: role-emotional, MH: mental health. (**B**) The mean of three summary scores of SF-36, physical component summary (PCS), mental component score (MCS) and role-social component score (RCS) are shown; 371.6, 480.6, and 357.7, respectively.

SF-36 scores were also obtained from all participants, and the average score of each domain is shown in Fig. 3A. The average score in each domain was 42.8 (physical functioning; PF), 43.4 (role-physical; RP), 50.9 (bodily pain; BP), 44.5 (general health perception; GH), 48.1 (vitality; VT), 47.1 (social functioning; SF), 45.3 (role-emotional; RE), and 49.4 (mental health; MH). The average score of each domain of SF-36 is defined as 50.0 in healthy Japanese individuals^26,27^, and therefore the scores in PBC patients were comparable to those in healthy individuals in two domains, BP and MH. Meanwhile, the scores in PF, RP, and GH were markedly lower. In Japan, a factor structure is different from those in Europe and the United States, and thus one summary score of SF-36 (role-social component score [RCS]) is used in Japan in addition to two original summary scores (physical component score [PCS] and mental component score [MCS])^27^. In this cohort, PCS, MCS, and RCS were 371.6, 480.6, and 357.7, respectively (Fig. 3B), suggesting that the HRQOL in Japanese PBC patients was substantially impaired in physical and role-social components.

Since significant associations were observed between PBC-40 and SF-36 in the original study^9^ and in our previous validation of the Japanese version^25^, we aimed to confirm this association in this cohort (Table 2). Significant correlations were observed between every combination of PBC-40 and SF-36 domain. In particular, moderate to strong correlations were observed between the PBC-40 fatigue domain and each domain of the SF-36, and also moderate correlations existed between the PBC-40 social domain and RP, GH, VT, SF, and RE domains of SF-36. By contrast, the PBC-40 itch domain had a relatively weak correlation with SF-36.

**Table 2:** Association of PBC-40 domains and SF-36 domains.

	PF	RP	BP	GH	VT	SF	RE	MH
Symptoms	−0.385	−0.460	−0.532	−0.467	−0.468	−0.443	−0.463	−0.368
Itch	−0.319	−0.333	−0.343	−0.318	−0.313	−0.343	−0.316	−0.233
Fatigue	−0.523	−0.607	−0.548	−0.578	−0.705	−0.537	−0.554	−0.510
Cognitive	−0.475	−0.539	−0.372	−0.419	−0.453	−0.408	−0.528	−0.420
Social	−0.470	−0.590	−0.454	−0.606	−0.579	−0.588	−0.509	−0.496
Emotional	−0.256	−0.361	−0.320	−0.531	−0.462	−0.411	−0.336	−0.482

Pearson correlation efficient is shown in the table.

PF: physical functioning, RP: role-physical, BP: bodily pain, GH: general health perception, VT: vitality, SF social functioning, RE: role-emotional, MH: mental health.

### Association of variables with PBC-40 fatigue and itch domain

Next, we focused on fatigue and pruritus, which could significantly impair the HRQOL of patients with PBC, and aimed to elucidate which clinical and laboratory variables were related to the development of these two symptoms. We employed age at diagnosis, follow-up period, age at participation, aspartate transaminase (AST), alanine transaminase (ALT), alkaline phosphatase (x upper limit of normal [ULN]), bilirubin, and albumin levels for the analysis. The correlation between the fatigue or itch scores and continuous variables are shown in Table 3A. The correlations of the fatigue score were significant with almost all variables except for age at participation, and those of itch were significant with age at diagnosis, follow-up period, alkaline phosphatase, and albumin levels. Regarding categorical variables, we examined sex, presence of clinical events, use of UDCA, and use of bezafibrate. Female sex, presence of events, and no use of UDCA were significantly associated with high fatigue scores, and only presence of events was associated with high itch scores (Table 3B).

**Table 3:** Association of clinical and laboratory variables with PBC-40 scores in fatigue and itch domain.

Variables		Fatigue domain		Itch domain	
		*R*	*P*	*R*	*P*
**A**. **Continuous variables**					
Age at diagnosis (years)		−0.137	**0**.**004**	−0.124	**0**.**009**
Follow-up period (years)		0.155	**0**.**001**	0.204	**<0**.**001**
Age at participation (years)		−0.038	0.395	0.027	0.545
AST level (U/L)		0.155	**0**.**001**	0.086	0.061
ALT level (U/L)		0.108	**0**.**018**	0.050	0.272
Alkaline phosphatase level (xULN)		0.221	**<0**.**001**	0.196	**<0**.**001**
Bilirubin level (mg/dL)		0.135	**0**.**003**	0.042	0.365
Albumin level (g/dL)		−0.238	**<0**.**001**	−0.105	**0**.**029**
**Variables**	**Categories**	**Fatigue domain**		**Itch domain**	
		**Mean (SD)**	***P***	**Mean (SD)**	***P***
**B**. **Categorical variables**					
Sex	Male (n = 53)	20.83 (7.26)	**0**.**001**	5.66 (2.76)	0.910
	Female (n = 443)	24.54 (8.89)		5.71 (3.02)	
Presence of events	Absent (n = 366)	23.60 (8.27)	**<0**.**001**	5.70 (3.00)	**0**.**033**
	Present (n = 49)	28.49 (10.10)		6.71 (3.07)	
Use of UDCA	Yes (n = 384)	24.07 (8.60)	**0**.**005**	5.83 (2.98)	0.530
	No (n = 51)	27.75 (10.13)		6.12 (3.59)	
Use of bezafibrate	Yes (n = 100)	24.04 (8.91)	0.558	6.13 (3.11)	0.330
	No (n = 335)	24.64 (8.86)		5.79 (3.04)	

R indicates Pearson correlation efficient. Bold text is used if P value is less than 0.05.

Bold text is used if P value is less than 0.05.

### Multivariate analysis

Finally, we performed multivariate analysis using a logistic regression model to predict the development of fatigue and pruritus after adjustment of confounders (Table 4). Regarding fatigue, female sex, younger age at diagnosis, and lower albumin levels (<3.5 g/dL) were identified as independently contributing factors after adjustment of other variables. Conversely, longer follow-up periods (>10 years) and lower albumin levels (<3.5 g/dL) were determined as risk factors for developing a higher score of itch.

**Table 4:** Multivariate analysis of the association between clinical and laboratory variables with fatigue and itch scores of PBC-40.

	β (SE)	Adjusted OR (95% CI)	*P*
**Fatigue domain**			
Sex (female)	1.42 (0.59)	4.13 (1.31–13.0)	0.016
Age at diagnosis (by 10 years)	−0.40 (0.13)	0.67 (0.52–0.87)	0.002
Albumin level (≤3.5 g/dL)	1.50 (0.54)	4.48 (1.57–12.8)	0.005
**Itch domain**			
Follow-up period (>10 years)	0.34 (0.15)	1.41 (1.04–1.90)	0.025
Albumin level (≤3.5 g/dL)	0.88 (0.42)	2.41 (1.05–5.53)	0.037

Fatigue domain: adjusted by follow-up periods, age at participation, presence of events, AST level, ALT level, alkaline phosphatase level, bilirubin level, UDCA use, and bezafibrate use. Itch domain; adjusted by sex, age at diagnosis, age at participation, presence of events, AST level, ALT level, alkaline phosphatase level, bilirubin level, UDCA use, and bezafibrate use.

**Figure 4:**
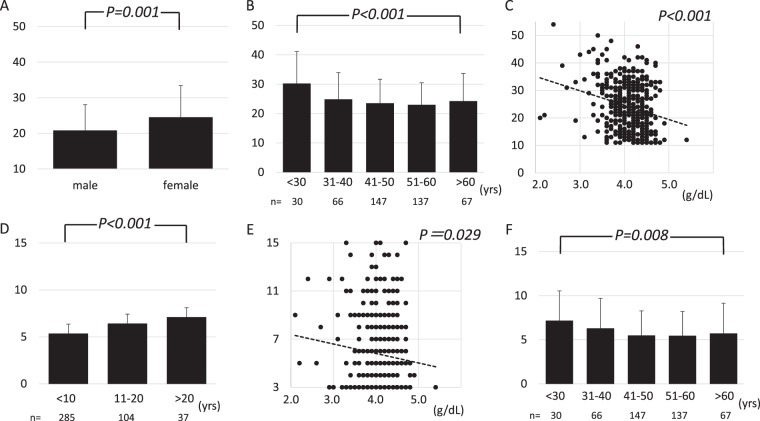
(**A**) The average of the PBC-40 fatigue score in males and females. The score was significantly higher in females compared to males (P = 0.001). (**B**) The average of the PBC-40 fatigue score depending on age at presentation is shown. Patients with younger age at presentation were likely to experience more fatigue (P < 0.001). (**C**), A scatter diagram with serum albumin level on the X-axis and the score in fatigue domain on the Y-axis, indicating a significant correlation between the serum albumin level and the fatigue score (P < 0.001). (**D**) The average of the PBC-40 itch score depending on follow-up period (years) is shown. Patients with a longer follow-up period were likely to experience more pruritus (P < 0.001). (**E**), A scatter diagram with the serum albumin level on the X-axis and the score in the itch domain on the Y-axis, indicating a significant correlation between serum albumin level and itch score (P = 0.029). (**F**) The average of the PBC-40 itch score depending age at presentation is shown. Patients with younger age at presentation were likely to experience more pruritus (P = 0.008).

In Fig. 4, we illustrate the association of fatigue scores with sex, age at diagnosis, and serum albumin level, and also the association of itch scores with follow-up periods, serum albumin levels, and age at diagnosis.

## Discussion

In the current study, we enrolled 496 Japanese patients with PBC, and measured symptoms and the HRQOL of these patients. All of the studies focusing on HRQOL in patients with PBC have been performed in Europe and the United States, and especially in the UK. This study was the first to evaluate symptom severity and HRQOL of PBC patients in an ethnic group other than Europe and North America. Since symptoms and HRQOL experienced by PBC patients may be greatly influenced not by only clinical phenotype affected by genetic backgrounds and environmental factors, but also by social and cultural difference among ethnic groups, the measured impact of PBC on HRQOL in European countries cannot be extrapolated to other countries with different ethnic, social, and cultural backgrounds. In this regard, the current study is of particular importance to elucidate the impact of PBC on HRQOL in different ethnic groups. The Japanese version of the PBC-40 translated and developed by our group was validated previously. It showed internal consistency in each domain (Cronbach’s alfa coefficient) ranged from 0.72 (symptom) to 0.95 (fatigue), and exploratory factor analysis demonstrated that various goodness of fit indices were well acceptable^25^. The correlation of PBC-40 with SF-36 was confirmed in the previous study^25^ and again in the current study (Table 2), indicating acceptable criterion-based validity.

We demonstrated that there is a substantial burden of symptoms and impaired HRQOL in Japanese patients with PBC. The proportion of patients who scored “moderate” and “severe” in each domain of the PBC-40 ranged from 24.0% (social) to 54.6% (emotional) (Fig. 2), indicating that half or at least one-fourth of Japanese PBC patients experienced a significant burden of the disease in daily life, irrespective of severity. In addition, the score of SF-36 also suggested that the HRQOL of Japanese PBC patients were intensively impaired, especially in physical and role-social components.

Of note, we also observed that the average scores for the PBC-40 for each domain in this study was lower than those in the UK studies. In the original PBC-40 report from the UK cohort, the score was highest in the fatigue domain (3.2), followed by emotional (3.0), social (2.9), symptoms (2.6), cognitive (2.6) and itch (2.0) domains^9^. Another study from the UK also indicated very similar results: fatigue (2.9), emotional (2.6), social (2.6), cognitive (2.5), symptoms (2.3), and itch (1.4)^13^. Thus, while the score in the current Japanese cohort was the lowest for fatigue, cognitive, social, and symptoms domains, the scores were comparable in the emotional and itch domains. The reason for this difference is unclear, and it remains to be determined which factor, i.e. genetic, geographical, social, or cultural factor, is mostly contributing to this difference. Currently, another international comparable study involving Japan, the UK, Spain, and Italy measuring HRQOL using identical questionnaires is ongoing and the results are awaited for the dissection of ethnical differences.

Despite these differences, it is of particular interest that determinants of symptom severity demonstrated in the current study were identical to those in the UK-PBC cohort, which indicated that sex and age were determinants of symptoms^10^. Younger age at diagnosis was associated with having more fatigue, after adjustment for age at participation, presence of events, and treatment protocol. Regarding pruritus, the follow-up period, and not age at presentation, was independently associated with the severity of pruritus. However, there was a strong correlation between age at presentation and follow-up period. The Pearson correlation efficient between age at presentation and follow-up period was −0.484 (p < 0.001), and the score in itch domain was significantly higher in patients with presentation at a younger age (p = 0.008) (Fig. 4F). Female sex was also identified as a risk factor for severe fatigue (Fig. 4A). In contrast, the severity of symptoms was not related to the use of UDCA. Collectively, PBC symptoms are primarily determined by the disease process itself, despite ethnic, cultural, and social differences, even though the strength of the symptoms represented by the PBC-40 scores seemed to be affected by these factors. Nevertheless, as an independent determinant for both pruritus and fatigue, we identified serum albumin levels, which are possibly associated with disease progression. This was in contrast to the findings in the UK cohort^12^.

Nevertheless, the current study presents several limitations. The sample size was smaller compared to recent studies in the UK^10–12^. Critical factors which were reported to be associated with symptoms in PBC patients, such as daytime somnolence, depression, anxiety, autonomic symptoms^10–12^, were not measured in the current study, as we wished to emphasize the feasibility of this study and hence, did not make the questionnaires too cumbersome. Further studies with enrolment of more Japanese PBC patients are warranted with similar questionnaires used in the UK studies. Another limitation in the current study was the lack of age- and sex- matched controls resulting in the impossibility to confirm whether the symptoms reported by PBC patients were really disease-specific. In this regard, PBC-40c^12^, designed and developed for measuring HRQOL in control individuals, should be translated, validated and used for HRQOL studies in Japanese. In contrast, a score 50 for SF-36 was defined as the average in healthy Japanese individuals, and therefore the decrease of SF-36 scores (Fig. 3) confidently indicates the impairment of HRQOL compared to healthy individuals. Finally, this study was a cross-sectional study and the outcomes of these patients are not currently known. Follow-up data for this cohort is urgently required to investigate whether patients who develop PBC at younger age are less likely to respond to UDCA and are at higher risk of progression, as resulted from the UK cohort^10^.

Life expectancy of PBC is favourable with the introduction of UDCA as a first-line drug. The use of bezafibrate in Japan might further improve the long-term outcome^28^. Therefore, it is extremely important to define improvement of HRQOL as another treatment target in PBC. In this regard, the current study, the first to evaluate symptoms and HRQOL in ethnicities other than European or North American, provides fundamental information to develop a therapeutic strategy for the improvement of symptoms and HRQOL. Furthermore, this study could contribute to aetiological consideration of symptoms in PBC, through comparison with HRQOL in the UK and in other areas. Additional international, comparative studies using an identical protocol are awaited.

## Patients and Methods

### Study design and subjects

This was a multicenter, observational, cross-sectional study, designed and conducted by the Japan PBC Study Group (JPBCSG), a branch of the Intractable Hepatobiliary Disease Study Group in Japan. Consecutive patients with PBC at the outpatient clinic of each center were invited to participate in this study between July 1, 2015, and October 31, 2016. Eleven centers in Japan participated in this study. All centers are tertiary, distributed to the whole area of Japan. Diagnosis of PBC was made with established criteria consisting of two or more of the following: chronic elevation of cholestatic liver enzymes, detectable AMA in sera, and compatible or diagnostic liver histology^29^. We obtained informed consents from all patients on the participation. We asked patients who agreed to participate in the study to fill out the questionnaires consisting of the Japanese version of PBC-40 and short-form 36 (SF-36) for the assessment of symptoms and HRQOL. In addition, we requested the Tokyo Hepatitis Association, a patient advocacy group for liver diseases, to collaborate in this study and to distribute the questionnaires to all members with PBC. Detailed clinical information was collected at participating centers. These data included the date of diagnosis, liver histological stage at diagnosis if liver biopsy was performed, liver biochemistry, and presence of the clinical events (i.e., jaundice, ascites, oedema, hepatic encephalopathy, oesophageal or gastric varices, and hepatocellular carcinoma). Patients were not invited to participate if they experienced admission during the study period due to any reason, had a history of liver transplantation because of PBC, had other aetiologies of liver diseases including autoimmune hepatitis. Additionally, those who had co-morbidities which had severe symptoms or poor outcomes and could greatly affect HRQOL were excluded, such as rheumatoid arthritis with severe pain, atopic dermatitis with severe pruritus, and malignant diseases. The current study was conducted in accordance with the ethical standards laid down in the Helsinki Declaration by the World Medical Association, as well as “Ethical guidelines for medical and health research involving human subjects” presented by the Ministry of Health, Labor and Welfare of Japan. The study protocol was approved by the Ethics Committee of Teikyo University (approval no. 14–186) and other participating centers.

### Symptoms and HRQOL assessment tools

For the assessment of symptoms and HRQOL in patients with PBC, we employed the Japanese version of PBC-40 and short form (SF)-36. PBC-40 is a patient-derived, disease-specific measure of symptoms and HRQOL, consisting of 6 domains; symptoms, itch, fatigue, cognitive, social and emotional^9^. Since the PBC-40 was originally developed in English, we translated the original version into Japanese and confirmed its acceptability, reliability, construct validity, and precision^25^. Each aforementioned domain included 7, 3, 11, 6, 10, and 3 items, respectively. Since scores in each item range from 1 to 5 points, the range of scores in each domain was 7 to 35, 3 to 15, 11 to 55, 6 to 30, 10 to 50, and 3 to 15, respectively. In the current study, we defined the severity of each domain into four categories as follows; “none” if the score was 0, “mild” if the score was between 0 and the one-third of the full score, “moderate” for scores between one-third and the two-thirds of the full score, and “severe” for scores between the two-thirds of the full score and the full score. The SF-36 Health Survey is a questionnaire used to measure health status in general. In the SF-36, one item is designed to assess perceived change in health status, and each of the remaining 35 items contributes to a score on one of eight scales: PF, RP, BP, GH, VT, SF, RE, and MH^30^. The Japanese version of SF-36 was already established and validated previously^26,27^.

### Statistical analyses

All statistical analyses were performed using SPSS^®^ Statistics version 22 (IBM Japan, Tokyo, Japan). Continuous variables are presented as mean (standard deviation) if normally distributed, or median (interquartile range) otherwise. We excluded the prothrombin time and international normalisation ratio for analysis since missing rates for these two variables were exceptionally high (47% and 50%, respectively). The Pearson correlation efficient was calculated for the assessment of the correlation between SF-36 and PBC-40, and between continuous variables and PBC-40 fatigue/itch domain scores. Comparison of fatigue/itch scores between two categories of sex, presence of events, use of UDCA/bezafibrate was performed using Mann-Whitney’s U test. Multivariate analysis was performed using a logistic regression model for adjustment of variables. Presence of fatigue or pruritus as dependent variables was binary and categorised as severe/moderate and mild/none as defined by the score. One-way ANOVA was used for comparison among three or more groups. A result was considered to be statistically significant when the *P* value was <0.05.
